# Armed conflict and household food insecurity: evidence from war-torn Tigray, Ethiopia

**DOI:** 10.1186/s13031-023-00520-1

**Published:** 2023-05-05

**Authors:** Aregawi Weldegebreal Weldegiargis, Haftom Temesgen Abebe, Hiluf Ebuy Abraha, Meron Micheale Abrha, Tsegay Berihu Tesfay, Rieye Esayas Belay, Alemnesh Abraha Araya, Mengish Bahresilassie Gebregziabher, Hagos Godefay, Afework Mulugeta

**Affiliations:** 1 School of Public Health, College of Health Sciences, Mekelle University, Tigray, Ethiopia; 2 College of Health Science, Ayder Comprehensive Specialized Hospital, Clinical Governance and Quality Improvement unit, Mekelle University, Tigray, Ethiopia; 3 Tigray Regional Health Bureau, Tigray, Ethiopia; 4 Tigray Health Research Institute, Tigray, Ethiopia

**Keywords:** Armed conflict, War, Hunger, Household Food insecurity, Tigray, Ethiopia

## Abstract

**Background:**

Exposure to armed conflicts result in strongly adverse and often irreversible short- and long-term effects which may transmit across generations. Armed conflicts directly cause food insecurity and starvation by disruption and destruction of food systems, reduce farming populations, destroying infrastructure, reducing resilience, and increasing vulnerabilities, disruptions in access to market, increasing food price or making goods and services unavailable altogether. The objective of the present study was to determine the status of household food insecurity in the armed conflict affected communities of Tigray in terms of Access, Experience and Hunger scale.

**Method:**

Community-based cross-sectional study was conducted to assess impact of armed conflict on household food insecurity among households with children with under one year. FHI 360 and FAO guidelines were used to quantify household food insecurity and Household hunger status.

**Results:**

Three-fourth of the households had anxiety about food supply and eat undesired monotonous diet due to lack of resources. Households were obliged to eat few kinds of foods, eat smaller meals, eat foods they do not want to eat, or went a whole day without eating any food. Household food insecurity access, food insecurity experience, and hunger scales significantly increased by 43.3 (95% CI: 41.9–44.7), 41.9 (95% CI: 40.5–43.3) and 32.5 (95% CI: 31.0-33.9) percentage points from the prewar period.

**Conclusions:**

Household food insecurity levels and household hunger status of the study communities was unacceptably high. The armed conflict has significant negative effect on food security in Tigray. It is recommended that the study communities need to be protected from the immediate and long-term consequences of conflict-induced household food insecurity.

## Background

The human costs of armed conflicts are enormous. Millions of lives are affected and lost. Evidence shows that exposure to armed conflicts result in strongly adverse and often irreversible short- and long-term effects which may transmit across generations [1–3]. In conflict-affected countries from sub-Saharan Africa, the number of undernourished people increased by 23.4 million between 2015 and 2018 – and at a faster rate compared to countries not exposed to conflict [4]. In addition to the humanitarian tragedies, armed conflicts directly cause food insecurity and starvation by disruption and destruction of food systems, reduce farming populations, destroying infrastructure, reducing resilience and increasing vulnerabilities, disruptions in access to market, increasing food price or making goods and services unavailable altogether [5, 6]. The worst food crises have occurred in areas of armed conflict illustrating a strong correlation between these variables [7]. For instance, about half of the people in Syria and Yemen are suffering from severe food insecurity [8, 9]. All nineteen countries classified by FAO as under “protracted crisis” conditions in 2017 were engaged in violent conflict at that time too [10]. Moreover, six out of ten countries with worst food crisis in the world in 2019 and all countries experienced famine in 2020 were caused due to conflict. It has also a spillover effect on food security in Ethiopia and Uganda [11].

The situation in the war-torn Tigray is not different. The armed conflict in Tigray region of Ethiopia erupted on the 4th of November 2020 had devastating consequences on the lives, health, and livelihoods of the people of Tigray. The armed conflict and its associated siege has led to death of civilians, massive displacement of residents, destruction of infrastructures, interruption of all government services such as banking, communication, health, education, transport, and others and near collapse of the economic activities in the region. Recent rapid field assessment by the Agriculture Bureau revealed that crops and animals were looted or destroyed, and the majority of the farmers were left without any food, seed, oxen, farm tools and farm inputs which led to the total collapse of the agriculture sector of Tigray [12]. Thus, the food shortages linked to the armed conflict would set the stage for years of food emergencies in Tigray, even after fighting ceases.

However, the level of the armed conflict linked household food insecurity and its aftermath is insufficiently studied in Tigray. High quality data on household food insecurity level is needed for improved targeting and programing. This study will give better quality results of the status of the households in Tigray and ultimately translate into better allocation of limited resources. Moreover, there is less evidence on how the armed conflict has affected the household food insecurity in Tigray. To a large extent, this is due to the difficulty to collect data during the periods of armed conflict and associated blockade, or siege imposed on Tigray. Finally, armed conflict and food insecurity data are needed to advance the discussion of causality and future lifesaving food security interventions in Tigray, Northern Ethiopia.

In this study, it is hypothesized that the armed conflict has seriously impacted the household food security of the study communities. Thus, the objective of the present study was to determine the status of household food insecurity in the armed conflict affected communities of Tigray in terms of Access, Experience and Hunger scale. Household food insecurity access scale (HFIAS) status was analyzed based on FHI-360 HFIAS indicator guide 2007 [13] and compared with the prewar status of the 2019 food insecurity level [14]. Household Hunger Scale (HHS) was analyzed based on FHI-360 HHS indicator definition and measurement guide, 2011 [15]. Household Food Insecurity Experience (HFIES) was done based on the FAO guide[16].

## Materials and methods

**Fig. 1:**
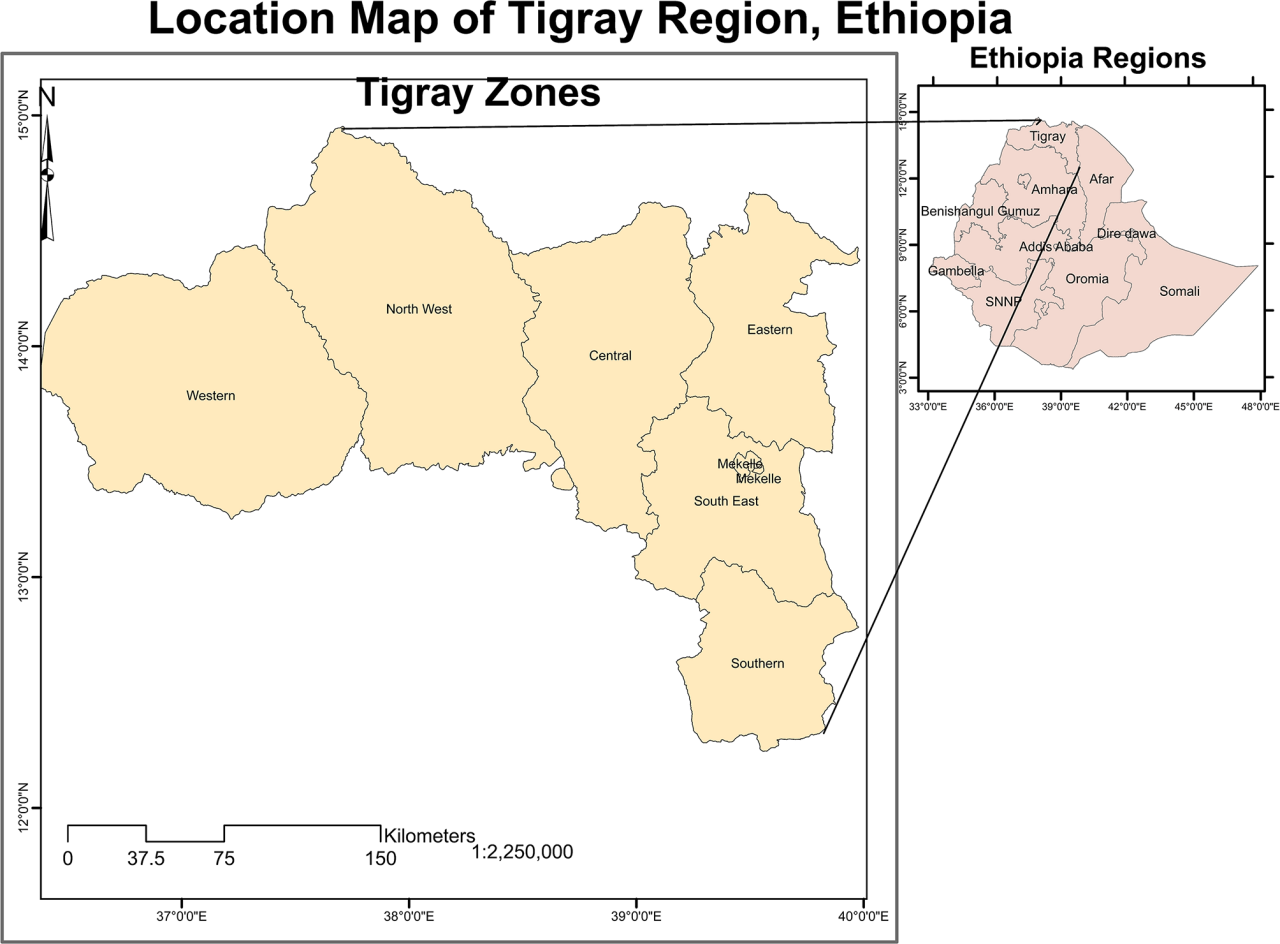
Location map of Tigray, Northern Ethiopia, 2023

Study setting: The household survey was conducted in randomly selected 52 districts of Tigray, Northern Ethiopia. Tigray is bordered by Eritrea, Sudan, Amhara Regional State and Afar Regional State from the North, West, South and East, respectively (Fig. 1). Administratively, it is divided into seven zones namely Central, Eastern, Mekelle, Northwestern, Southern, Southeastern and Western zones. The districts from the Western zone of Tigray were not included in the study for security reasons. The survey was conducted in July and August 2021. Study design: A community based cross sectional survey was conducted to collect data on household food security.

Study population: The study units were households with under one years of age children and the study populations were women or caretakers of under one years of age children. Data for this study were extracted from a larger survey collected on key performance indicators for health and nutrition of children during the war period.

Sample size and sampling procedure: A total of 52 districts were randomly selected from six administrative zones of Tigray except the Western zone. Form each district, four Tabias (smaller administrative units) were randomly selected and from each Tabia, 20 households with under one years of age children were selected randomly using the household list of the health extension workers as a sampling frame. The sample size was calculated to be 4160 (52 districts * 4 Tabias/district * 20 households/Tabia). Adding 5% of non-response, the total minimum sample size was determined to be 4368.

Data collection: The data collection tool for assessing the household food insecurity was adapted from the indicator guide for Household Food Insecurity Access Scale (HFIAS) for Measurement of Household Food Access [13]. The tool was translated from English to Tigrigna (the local language) and then back to English to ensure consistency. Data collectors were trained on the purpose of the study, data collection methods and other practical issues. Supervisors experienced in similar surveys data collection were assigned per each study site to monitor the data quality. On top of this, the completed tools were checked for completeness each day of the data collection.

### Food insecurity measurements

#### HFIAS measures

Field validation studies have demonstrated the feasibility and usefulness of HFIAS measures in developing country contexts. The HFIAS measures are constructed based on households’ experience of food insecurity. The constructed measures were found to be strongly correlated with the indicators of poverty, food consumption and food security related activities. The measures are also sensitive to the changes in the household situation over time. Moreover, the set of the nine HFIAS questions appear to distinguish between food secure and insecure households across different cultural contexts in several countries. This makes the HFIAS measures valid and useful for assessing the impacts of the armed conflict on household food insecurity in Tigray, Northern Ethiopia.

Each question was asked with a four-week or 30-days recall period. First, the respondent was asked whether the condition in the question happened at all in the past four weeks: the occurrence question (yes or no). If the answer to the occurrence question was “yes”, then the frequency-of-occurrence was asked to determine whether the condition happened rarely (once or twice), sometimes (three to ten times), or often (more than ten times) in the past four weeks. The “no” and “yes” responses were recorded as 0 and 1, respectively. Similarly, the rarely, sometimes, and often responses were recorded as 1, 2 and 3, respectively. Therefore, a given household can score between 0 and 27 on the frequency of occurrence to the nine HFIAS questions. households with 0–1, 2–13, 14–16 and 17 and above scores were categorized as food secure, mildly food insecure, moderately food insecure and severely food insecure, respectively.

### HFIES Measures

In the household food insecurity experience scale the questions are eight like the HFIAS excluding one question. The frequency-of-occurrence questions are excluded. The score in HFIES ranges between zero and eight. The lower the score the household has experienced lower or no food insecurity and the higher the score, the household experienced severe levels of food insecurity. Households with zero, 1–3, 4–6, and 7–8 scores were categorized as having experienced no food insecurity, mild food insecurity, moderate food insecurity and severe food insecurity (hunger), respectively.

### HHS Measures

Household Hunger scale is constructed from the last three questions of the nine HFIAS questions (Q7, Q8 and Q9). The frequency-of-occurrence questions were used to estimate the level of hunger in the households assessed. The total scores in HHS range between zero and six. Households with 0–1, 2–3 and 4–6 scores were classified as having little or no hunger, moderate hunger, and severe hunger, respectively. Data analysis: Data was entered into Epi Data software version 3.1 and then transferred into SPSS version 25 software for analysis. Descriptive analysis was done, and the processed data was presented using tables and figures. To compare pre and war period food security conditions, we used the test for equality of proportions.

Ethical clearance: The study was ethically cleared as part of a larger survey on the Key Performance Indicators by the Institutional Review Board of the College of Health Sciences of Mekelle University (MU-IRB 1906/2021). Besides support letter from Tigray Health Bureau, District Health Offices and verbal consent from the study participants was secured prior to the actual data collection. The respondents’ confidentiality was maintained, and the names of the participants were not labeled.

## Results

The study was conducted in six of the seven zones of Tigray. The Western zone of Tigray was excluded for security reasons. A total of 4375 household respondents with a response rate of 100% were included in the study. About 75.5% (3304) were female respondents and 84.5% of the households were food insecure [Table 1].

**Table 1:** Socio demographic characteristics of the study participants from the study communities in Tigray, Northern Ethiopia, 2021(n = 4375)

Variables	Category	Food insecure, n (%)	Food secure HH, n (%)	Total, n (%)
Sex	Female	2841(76.8)	464 (68.4)	3304 (75.5)
	Male	858 (23.2)	214 (31.6)	1071 (24.5)
	Total	3699 (84.5)	678 (15.5)	4375 (100)
Zone	Central	972 (26.2)	181 (26.9)	1153 (26.4)
	East	1146 (31.0)	78 (11.6)	1224 (28.0)
	Mekelle	260 (7.0)	37 (5.5)	297 (6.8)
	Northwest	599 (16.2)	94 (13.9)	693 (15.8)
	South	392 (10.6)	141 (20.9)	533 (12.2)
	Southeast	332 (9.0)	143 (21.2)	475 (10.9)

### Household food insecurity conditions

About 73.3% (3205) of the respondents reported experiences of anxiety and uncertainty about the household food supply in the four weeks (30 days) prior to the date of data collection. Similarly, 70.5% of the households were not able to eat according to their preference and 75.8% were able to eat an undesired monotonous diet due to lack of resources. Nearly 63.2% of the households had to eat food that they found socially or personally undesirable and close to three fourths (71.7%) of the respondents felt that the amount of food any household member ate in any meal during the past four weeks was smaller than they felt they needed. More than two thirds (69.8%) of the households had to eat fewer meals than the number typically eaten in the food secure households in their area and close to half (46.4%) of the households had no food to eat of any kind in their home. About 31.2% of the respondents felt hungry at bedtime and a quarter (26.0%) of the households reported that they did not eat from the time they awoke in the morning to the time they awoke the next morning due to lack of food. Statistically significant declines (p < 0.001) of household food security were observed in anxiety and uncertainty about the household food supply, insufficient quality (variety and preferences of the type of food) and insufficient food intake and its physical consequences in 2019 and 2021 (Table 2).

**Table 2:** Prewar (n = 11,004) and war period (n = 4375) comparison of food insecurity access-related conditions of the households in accessible study areas of Tigray, Ethiopia, 2021

Variables	Prewar 2019 (N = 11,004) n (%)	War period 2021 (N = 4375) n (%)	% Points difference (95% CI)	P-value
Worry about food	3908 (35.5)	3210 (73.3)	37.9 (36.3–39.4)	< 0.001
Unable to eat preferred foods	4058 (36.9)	3085 (70.5)	33.6 (31.9–35.2)	< 0.001
Eat just a few kinds of foods	3633 (33)	3317 (75.8)	42.8 (41.2–44.3)	< 0.001
Eat foods they really do not want to eat	2280 (20.8)	2767 (63.2)	42.5 (40.8–44.1)	< 0.001
Eat a smaller meal	1677 (15.2)	3137 (71.7)	56.5 (55.0-57.9)	< 0.001
Eat fewer meals in a day	1037 (9.4)	3053 (69.8)	60.4 (58.9–61.8)	< 0.001
No food of any kind in the household	464 (4.2)	2033 (46.4)	42.3 (40.7–43.8)	< 0.001
Go to sleep hungry	430 (3.9)	1367 (31.2)	27.4 (25.9–28.8)	< 0.001
Go a whole day and night without eating	277 (2.5)	1138 (26.0)	23.5 (22.1–24.8)	< 0.001

### Household Food insecurity (Access) scale

**Fig. 2:**
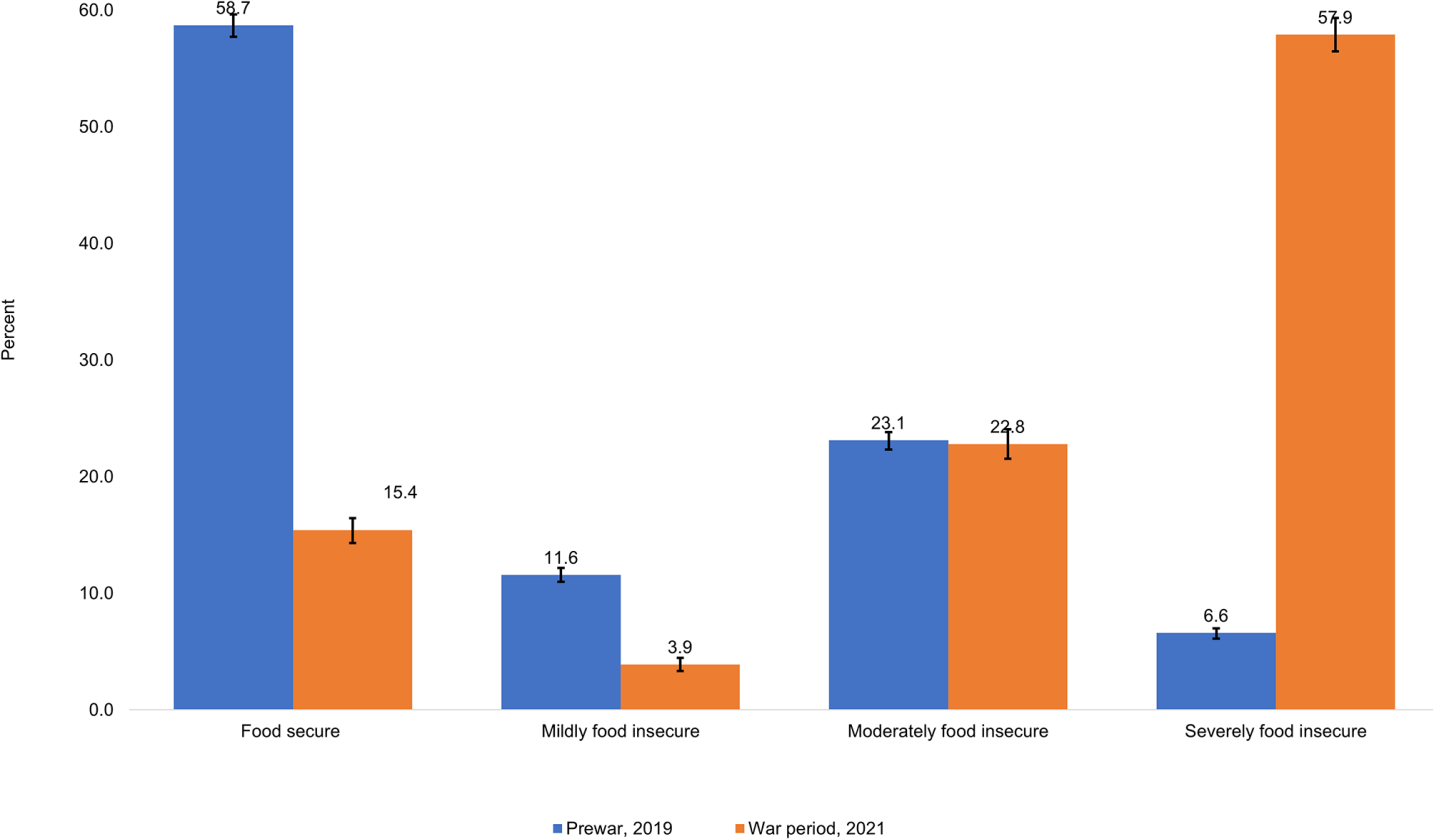
Prewar (n = 11,004) and war period (n = 4,375) comparison of household food insecurity (access) in the study communities of Tigray, Ethiopia, 2021

According to the Household Food Insecurity Access Scale (HFIAS), 15.4% (95% CI: 14.3–16.4) and 84.6% (95% CI: 83.5–85.6) of the households were food secure and food insecure in 2021 (war period) compared to 58.7% (95% CI: 57.7–59.6) and 41.3% (95% CI: 40.4–42.2) in 2019 (prewar), respectively (Fig. 2).

### Household Food insecurity (experience) scale

**Fig. 3:**
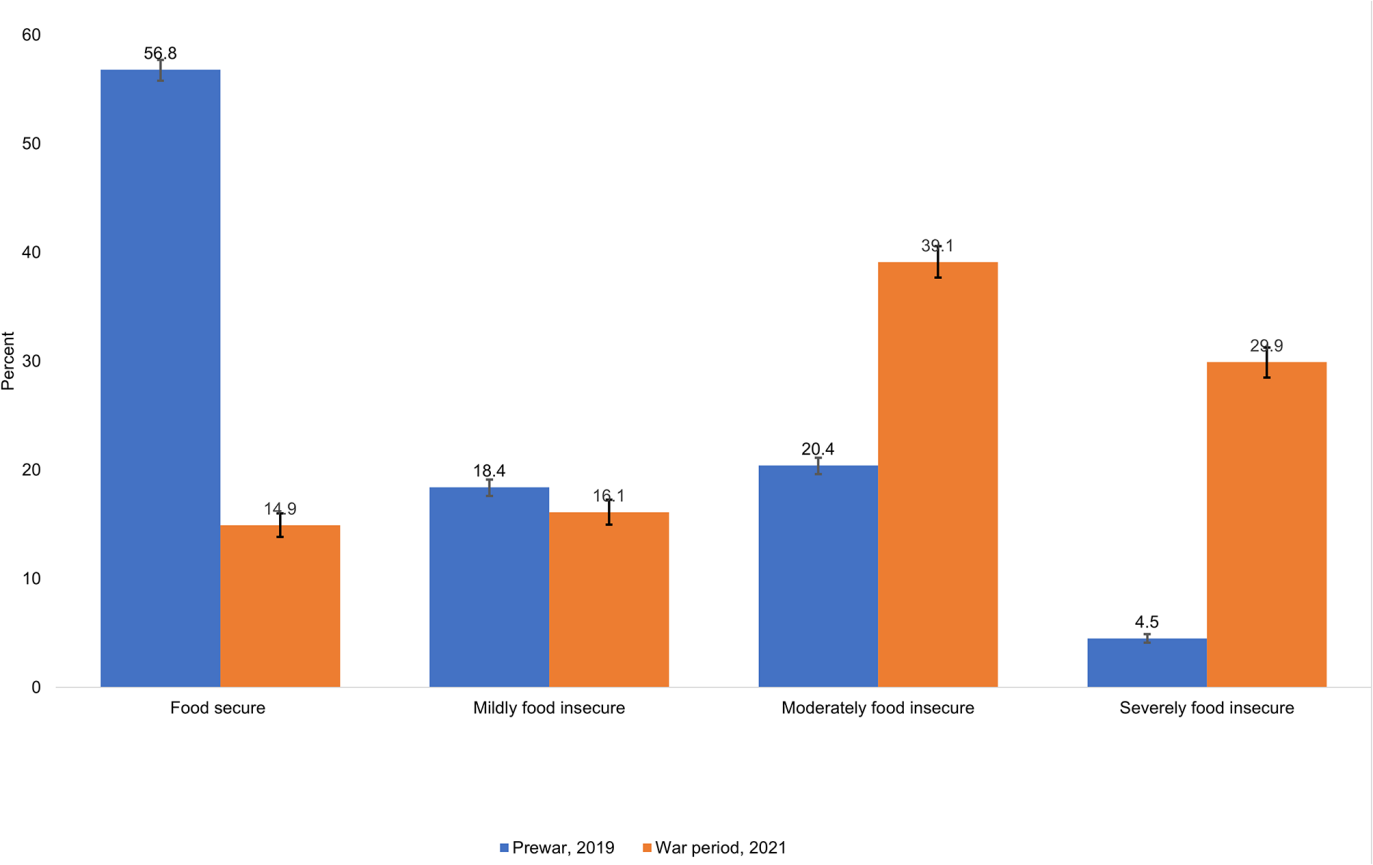
Prewar (n = 11,004) and war period (n = 4,374) comparison of household food insecurity (experience) in the study communities of Tigray, Ethiopia, 2021

The household food security experience (HFIES) is consistent with the access scale, but the severity of food insecurity experiences slightly differ. The percentage of households who experienced mild, moderate, and severe food insecurity were 16.1% (95% CI: 15.0-17.3), 39.1% (95% CI: 37.7–40.6) and 29.9% (95% CI: 28.4–31.2), respectively. Households who experienced mild, moderate, and severe food insecurity were increased by 41.9% points (95% CI: 40.5–43.3) from the 2019 prewar period. Households that experienced severe food insecurity had increased by 25.4% points (95% CI: 24.9–26.8). This increment was statistically significant at p < 0.001(Fig. 3).

### Household (Hunger) Scale

**Fig. 4:**
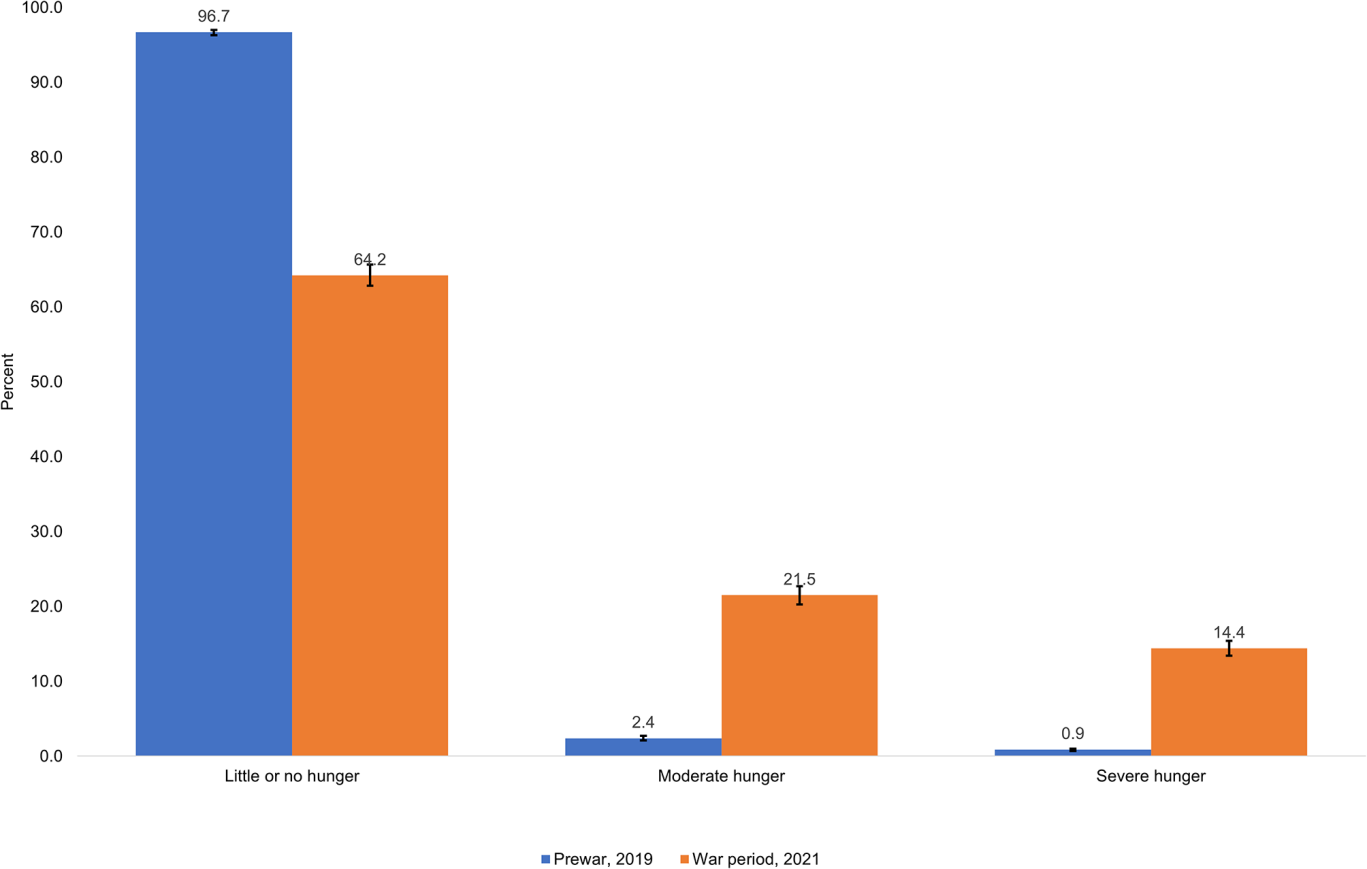
Prewar (n = 11,004) and war period (n = 4,371) comparison of household hunger in the study communities of Tigray, Ethiopia, 2021

According to the household hunger scale (HHS), 3.3% (95% CI: 2.9–3.6) of the households had moderate or severe hunger during the prewar period compared to 35.9% (95% CI: 34.2–37.2) during the war period corresponding to an absolute increase of 32.5% points (95% CI: 31.0-33.9) and the differences are statistically significant at P < 0.001 (Fig. 4).

### Household food insecurity score

The highest and minimum household food insecurity scores were observed in Northwest and Southeast zones of Tigray, respectively. Specifically, the highest score of severe food insecurity (Access), and severe food insecurity (Experience) were observed in 64.8%, 41.0% of the households from Northwest zone, respectively and the highest score of household hunger were observed in 22% of the households from Central zone of Tigray. Similarly, the minimum scores of severe food insecurity (Access), and severe food insecurity (Experiences) were observed in 40.2% and 12.1% of the households from southeast, respectively and the minimum scores of hunger scale were observed in 5.4% of the households from Mekelle zone of Tigray, (Table 3). The Spearman’s rho correlations among the variables HFIAS, HFIES, and HHS were also positively statistically significant at p < 0.001.

**Table 3:** Household Food Insecurity Access, Experience and Hunger Scale Score of the households in the study communities of Tigray, Northern Ethiopia, 2021 (N = 4377)

Household Food Insecurity Access Scale Score					
Zone	N	Food Secure, n (%)	Mild food insecurity, n (%)	Moderate food insecurity, n (%)	Severe food insecurity, n (%)
Central	1153	181 (15.7)	31 (2.7)	203 (17.6)	738 (64.0)
East	1224	78 (6.4)	39 (3.2)	326 (26.6)	781 (63.8)
Mekelle	297	37 (12.5)	16 (5.4)	113 (38.0)	131 (44.1)
Northwest	693	94 (13.6)	30 (4.3)	120 (17.3)	449 (64.8)
South	533	141 (26.5)	26 (4.9)	125 (23.5)	241 (45.2)
Southeast	475	143 (30.1)	29 (6.1)	112 (23.6)	191 (40.2)
Total	4375	674 (15.4)	171 (3.9)	999 (22.8)	2531 (57.9)
Household Food Insecurity (Experience) Scale Score					
Central	1152	174 (15.1)	145 (12.6)	364 (31.6)	469 (40.7)
East	1224	78 (6.4)	183 (15.0)	625 (51.1)	338 (27.6)
Mekelle	297	41 (13.8)	83 (27.9)	137 (46.1	36 (12.1)
Northwest	693	94 (13.6)	88 (12.7)	227 (32.8)	284 (41.0)
South	533	127 (23.8)	105 (19.7)	210 (39.4)	91 (17.1)
Southeast	475	137 (28.8)	102 (21.5)	148 (31.2)	88 (18.5)
Total	4374	651 (14.9)	706 (16.1)	1711 (39.1)	1306 (29.9)
Household (Hunger) scale Status					
Zone	N	Little to No Hunger, n (%)		Moderate Hunger, n (%)	Severe Hunger, n (%)
Central	1149	620 (54.0)		275 (24.0)	253 (22.0)
East	1224	798 (65.2)		267 (21.8)	159 (13.0)
Mekelle	297	240 (80.0)		41 (13.8)	16 (5.4)
Northwest	693	373 (53.8)		197 (28.4)	123 (17.7)
South	533	411 (77.1)		79 (14.8)	43 (8.1)
Southeast	475	364 (76.6		78 (16.4)	33 (6.9)
Total	4371	2806 (64.2)		938 (21.5)	627 (14.3)

## Discussion

In this study, the level of food insecurity (access and experience) and hunger status of the armed conflict affected households is analyzed and compared with the prewar status of food insecurity and hunger of the study communities from Tigray, Northern Ethiopia. Our findings revealed that 15.4% and 84.6% of the households were food secure and food insecure, respectively, based on HFIAS scale. Similarly, 14.9% of the households were food secure whereas 85.1% of the households suffered from food insecurity based on the HFIES scale. More than one in three (35.8%) of the households suffered from moderate or severe hunger and 64.1% of the households had little or no hunger, according to the HHS scale.

The high burden of households’ food insecurity in our study communities is in line with the assessment done by the World Food Program (WFP) where 83% of the households were food insecure [17], the IPC report with 70% household food insecurity [18] and with other adverse food security outcomes of armed conflict [19, 20]. The increased prevalence of household food insecurity and increment of food insecurity conditions were primarily due to the disrupted production and economic activities of the households attributed to the armed conflict. According to the Bureau of Agriculture and Rural Development of Tigray, 75% of the livestock were slaughtered or looted, all poultry out-growers were interrupted, 85% of all milk processes were disrupted and 65% of forage processors have become dysfunctional implying that the armed conflict exacerbated household food insecurity in the study communities [21].

Present study, the level of concern and lack of access to, variety and/or quantity of food were analyzed. Individuals have experienced a reduction in food quality, variety, or quantity because of insufficient resources. Majority of the households reported that they were worried that enough food may not be available in the household and were not able to eat preferred foods. Moreover, households were obliged to eat few kinds of foods, eat smaller meals and foods they do not want to eat. Quite a significant proportion of households had no food of any kind to eat and went a whole day and night without eating any food suggesting the severity of the armed conflict induced household food insecurity. The armed conflict remained the major driver of the alarming situation, especially in households who had lost access to their livelihoods. Among the reasons for the high levels of food insecurity might be the lack of physical and economic access to sufficient food in the study communities due to the armed conflict and siege. The high cost (unaffordability) of foods and persistently high levels of war induced poverty continue to keep foods out of reach for the people in Tigray. Thus, unfettered humanitarian access and local price controls can be considered as short-term remedies to improve food access and fragility of the food systems in the study communities in Tigray, Northern Ethiopia.

When the data is further disaggregated by zone, the worst food security situation was observed in the households from northwest zone. These findings were in line with the highest prevalence of food insecurity as revealed by the Emergency Food Security Assessment Report of Tigray [17]. The Northern west zone is one of the zones of Tigray where off farm income generating activities are available. Thus, the high burden of food insecurity in northwest zone of Tigray might be due to the war associated disruption of the off-farm activities in the northwest zone of Tigray. The highest food insecurity in a zone where it used to be better off before the war suggested the armed conflict had affected the off-farm income generating activities, which households used as income leveling strategy [12]. Thus, the intensity of the armed conflict coupled with the disruption of household off-farm income generating activities has exposed the people in the northwestern to high levels of food insecurity during the war period.

The armed conflict induced loss of income and disruption of the agricultural production and off-farm income generating activities have primarily impacted the food systems in the study communities through their negative effects on people’s access to food, including the unaffordability of foods. The unaffordability of foods might be a result of the effects of other drivers or factors on people’s income and on the cost of foods throughout the food system. The unaffordability of foods combined with low incomes, explain why the households in the study communities were not able to afford even the cheapest food. Furthermore, the household’s food insecurity was exacerbated by the loss of employment and total siege imposed on Tigray.

The high levels of household hunger status in the study communities (36%) was lower than the 44% of severe household hunger status in central clusters in Tigray [18]. Similarly, the proportion of households with no or little hunger (64%) was higher compared to the 45% of WFP [17]. These differences could be due to differences in methodologies as the IPC and WFP employed computer-assisted telephone interviews (CATI), as well as the hard to reach assessment method. The increment in the emergency food security assessment done by WFP could be due to the continuous blockade in Tigray.

The effects of conflict-induced household food insecurity are both immediate and long-term. If children failed to get the right nutrition in the first 1000 days (between conception and the second birthday), they are more likely to be stunted at 2 years of age, with lifelong consequences for their health, education and productivity [22, 23]. For instance, children in Burundi and Zimbabwe who experienced violent conflicts were significantly shorter (stunted) than others, affecting their health, education, and productivity throughout their lives [1, 24].

The high household food insecurity life threatening situation can be explained by the presence of war that effectively reduced food production, reduced food imports, raised food prices and unaffordability of foods implying that the armed conflict was the major driver of acute food insecurity in Tigray. Amongst the strengths of the study were the use of large sample size for generalizability of the study findings, use of HFIAS (access), HFIES (Experiences), and HHS to determine food insecurity and conducting the study immediately after the first round of the armed conflict which helped to reduce recall bias. The exclusion of the western zone of Tigray from this study for security reasons was one of the limitations of the study. There were also limitations associated with the measures as most valid cross-cultural tools de-emphasize some cultural specificity. Hence, the HFIAS, HFIES and HHS might not be the most sensitive measurement tools of food insecurity to use in every context.

## Conclusions

Household food insecurity levels and household hunger status of the study communities was unacceptably high. About 15.4% and 84.6% of the households were food secure and food insecure respectively. the empirical results revealed that the armed conflict has significant negative effect on food security in Tigray. Thus, the households in Tigray need to be protected from the immediate and long-term effects of conflict-induced household food insecurity though provision general monthly food rations to nearly all the people of Tigray, imposing price controls to keep prices stable, resumption of essential government services and allow food imports to increase supplies in Tigray. In the long term, the people should be supported to ensure that they have both physical and economic access to sufficient food at all times to meet their dietary needs for a productive and healthy life.

## Data Availability

Data set is available based on request.

## Abbreviations

CATI: Computer-assisted Telephone Interviews
FAO: Food and Agriculture Organization of the United Nations
HFIAS: Household Food Insecurity Access Scale
HFIES: Household Food Insecurity Experience Scale
HHS: Household Hunger scale
IPC: Integrated Food Security Phase Classification
WFP: World Food Programme of the United Nations
